# An Inhibitory Medial Preoptic Circuit Mediates Innate Exploration

**DOI:** 10.3389/fnins.2021.716147

**Published:** 2021-08-23

**Authors:** Jia Ryoo, Seahyung Park, Daesoo Kim

**Affiliations:** Department of Biological Sciences, Korea Advanced Institute of Science and Technology (KAIST), Daejeon, South Korea

**Keywords:** medial preoptic area, periaqueductal gray, GABAergic neuron, reinforcement, exploration

## Abstract

Animals have an innate motivation to explore objects and environments with unknown values. To this end, they need to activate neural pathways that enable exploration. Here, we reveal that photostimulation of a subset of medial preoptic area (MPA) neurons expressing the vesicular-GABA transporter gene (vgat+) and sending axonal projections to the ventrolateral periaqueductal gray (vPAG) increases exploration in a chamber but causes no place preference when tested there without photostimulation. Photoinhibition of MPA^vgat^–vPAG projections leads to no emotional changes as measured by normal activity in an open field assay. Electrophysiological recordings revealed that most GABAergic vPAG neurons are inhibited by MPA^vgat^ neurons. In contrast to a previous report that suggested that MPA^vgat^–vPAG neurons may impart positive valence to induce place preference, our results suggest that these neurons can increase innate exploration.

## Introduction

In nature, animals explore the environment for their survival. Such exploration is required for finding food, prey, monitoring for predators, and investigating males, females, and offspring. Various types of motivated behavior can lead to exploratory behavior, such as hunting, consuming, aggression, reproduction, parental care, and defensive behavior.

Exploration can be broadly divided into two types: extrinsic exploration is driven by the completion of a goal, such as food to satiate hunger or scanning for predators, whereas intrinsically driven exploration occurs due to non-homeostatic or reproductive drives and has been speculated to be curiosity and novelty-seeking driven, driving investigation of objects, stimuli, and environments for its own sake (Hughes, 1997).

Although studies addressing extrinsic exploration toward goals such as feeding (Viskaitis et al., 2017; Tryon and Mizumori, 2018; Hao et al., 2019), predation (Han et al., 2017; Li et al., 2018; Park et al., 2018; Shang et al., 2019; Zhao et al., 2019), and defensive behavior (Tovote et al., 2016; Evans et al., 2018; Li et al., 2018; Rozeske et al., 2018; Zhang et al., 2021) have been conducted, those that address intrinsic exploration have been lacking.

Studies that have attempted to elucidate the mechanisms underlying intrinsic exploration have typically involved inducing altered states of arousal or anxiety and observing the effects that such manipulations have on exploratory behavior. Such research includes the use of drugs such as methamphetamines, amphetamines, and methylphenidate (Berlyne et al., 1966; Dyne and Hughes, 1970; Robbins and Iversen, 1973) to alter states of arousal and the use of benzodiazepines and chlordiazepoxide (Hughes, 1972; Russell, 1973; Crawley et al., 1981; Holmes and Rodgers, 1999) to alter anxiety. Although such studies help to identify possible relationships between brain states and intrinsic exploration, the brain regions and neural circuitry underlying such phenomena have received little attention.

The medial preoptic area (MPA) is part of the anterior hypothalamus and has been implicated in playing a role in many types of innately motivated behavior, such as hunting (Park et al., 2018), anxiety (Zhang et al., 2021), reproductive (Wei et al., 2018; McHenry et al., 2017), and parental behavior (Wu et al., 2014; Kohl et al., 2018; Li et al., 2019; Zhang et al., 2021). Although such studies have also shown to a degree the capability of the MPA to increase extrinsic exploration, due to alterations in underlying homeostatic drives or reproductive drives, the potential role of the MPA in modulating intrinsic exploration has received little attention. A recent study (Zhang et al., 2021) showed that optogenetic activation of vgat neurons in the MPA is capable of increasing time spent in the stimulated side of a real-time place preference (RTPP). However, this experiment by itself fails to distinguish between intrinsic and extrinsic exploration, since changes seen in RTPP could be driven either due to changes in exploratory behavior itself or due to a reinforcing effect. Since exploration induced by motivational drives of the MPA would be expected to be associated with either aversive or appetitive valences, we sought to distinguish between extrinsic and intrinsic exploration by validating whether any changes in exploratory activity were accompanied by driving reinforcement through the use of conditioned place preference (CPP) tests. Thus, to this end, we employed optogenetics to specifically modulate vgat neurons of the MPA and validate if such modulations alter intrinsic exploration.

## Materials and Methods

### Animals

The animal study was conducted according to the Korean Advanced Institute of Science and Technology (KAIST) Guidelines for the Care and Use of Laboratory Animals and were approved by the Institutional Animal Care and Use Committee (Protocol No. KA2020-63). Vgat-ires-*Cre* (mixed background, Jackson lab, 016962), aged 7–8 weeks, were maintained under a 12-h light/dark cycle with *ad libitum* access to food and water. Both sexes of mice for behavioral experiments were group housed (three to five mice per cage), except for when they were isolated before a home cage test. Behavioral experiments were conducted 3–4 weeks after virus injection and surgical procedures. For photostimulation experiments, we used six mice to photoactivate MPA^vgat^ neurons and four mice as controls. Seven mice were used to photoactivate PAG-projecting MPA^vgat^ neurons and three mice as controls. Seven mice were used for photoinhibition of PAG-projecting MPA^vgat^ neurons and four mice as controls.

### Virus Injection and Surgical Procedures

Mice were anesthetized with 2,2,2-tribromoethanol (Avertin 2.5%) via intraperitoneal injection and placed on a motorized stereotaxic frame (Neurostar, Tübingen, Germany). AAV2/9.EF1α.DIO.hChR2 (H134R).mCherry (Addgene, Watertown, MA, United States) was stereotaxically injected unilaterally into the MPA (Bregma AP, +0.2 mm; ML, ± 0.3 mm; DV, −5.2 mm) of *vgat*-ires-*Cre* mice. Fiber optic cannulas (200 μm diameter; Doric Lenses, Quebec, QC, Canada) were implanted over the MPA (Bregma AP, 0.2 mm; ML, +0.3 mm; DV, −5.1 mm) or into the vPAG (Bregma AP, −4.7 mm; ML, +0.3; DV, −2.5 mm) in all mice injected with ChR2 constructs. AAV2.EF1α.DIO.mCherry (University of North Carolina Vector Core, Chapel Hill, NC, United States) were injected as control. For photoinhibition of MPA neurons, AAV2/9.EF1α.DIO.eNpHR3.0.eYFP (University of Pennsylvania, Philadelphia, PA, United States) was injected bilaterally into the MPA of *vgat*-ires-*Cre* mice. Fiber-optic cannulas containing dual optic fibers within a single cannula (200 μm diameter) were implanted into mice injected with NpHR constructs. AAV2/5.EF1α.DIO.eYFP (University of North Carolina Vector Core, Chapel Hill, NC, United States) were injected as control. For whole-cell patch-clamp recordings, AAV2/5.EF1α.DIO.eYFP (University of North Carolina Vector Core, Chapel Hill, NC, United States) was also injected unilaterally into the vPAG (Bregma AP, −4.7 mm; ML, +0.3; DV, −2.4 mm) of *vgat*-ires-*Cre* mice. A total of 0.5 μl of each virus was injected at the following titers: 3.38 × 10^12^ genomic copies/ml for AAV2/9.EF1α.DIO.hChR2 (H134R).mCherry; 5.70 × 10^12^ genomic copies/ml for AAV2.EF1α.DIO.mCherry; 2.30 × 10^13^ genomic copies/ml for AAV2/9.EF1α.DIO.eNpHR3.0.eYFP; and 6.50 × 10^12^ genomic copies/ml for AAV2/5.EF1α.DIO.eYFP. Fiber-optic cannulas were implanted over the MPA and vPAG, secured to the skull with adhesive cement (Sun Medical, Shiga, Japan) and covered with dental cement (Vertex, Zeist, the Netherlands). All mice were allowed to recover for 3–4 weeks before any behavioral experiments were performed.

### Behavioral Experiments

All behavioral tests were conducted in a sound attenuation booth during the dark cycle, and all mice were handled for 10 min each day for 5 days before performing any test, except RTPP/CPP tests. The mice had an interval of 72 h between experiments. All mice were evaluated for viral expression and excluded if they lacked proper expression or implantation of fiber optic cannula with reference to a brain atlas (Paxinos and Franklin, 2008). No sex-dependent changes were observed; thus, male and female mice were pooled together.

#### Open Field Test

Mice were habituated in an open field test chamber (40 cm × 40 cm × 40 cm) for 10 min. The experiment was carried out for a total of 9 min. Sessions were conducted as light OFF–ON–OFF, each session lasting for 3 min. The locomotion of mice was recorded with a camera to measure distance movements (Kalueff et al., 2006).

We use the Gaussian mixture model to analyze locomotive patterns. To analyze locomotive patterns, we plotted the density of the log speed and used a Gaussian mixture model to analyze the distribution of these log speeds. The parameters of the model are estimated by using the Expectation–Maximization (EM) algorithm. The algorithm estimates the maximum likelihood parameters (mean, variance, and weights) of a mixture with a given number of clusters. We used three clusters: lingering, progressing, and rapid movement (fast progressing). The EM algorithm is an iterative algorithm that starts with user-given initial values and incrementally improves the likelihood function until further iterations yield only a negligible improvement (Drai et al., 2001).

#### Attached Object Test

Mice were habituated in a test chamber (40 cm × 40 cm × 40 cm) for 10 min. After habituation, an object (2 cm × 2 cm × 2 cm) was attached to the center of the chamber. The experiment was carried out for a total of 9 min. Sessions were conducted as light OFF–ON–OFF, each session lasting for 3 min. The locomotion of mice was recorded with a camera to measure frequency of approaching toward the object zone (20 cm × 20 cm). Linear regression was done by using Microsoft Excel.

#### Home Cage Test

Mice were habituated in their home cage for 10 min while connected to an optic cable. The experiment was carried out for a total of 9 min. Experiments were conducted as sessions of light OFF–ON–OFF, with each session lasting for 3 min. The locomotion of mice was recorded with a camera to measure rearing behavior.

#### Real-Time Place Preference and Conditioned Place Preference Test

The RTPP and CPP apparatus consisted of two rectangular chambers (20 × 18 cm^2^) with distinct wall drawings and a corridor separating them. One rectangular chamber had a polka dot pattern, and the other rectangular chamber had a striped pattern. A video-tracking and analysis system (EthoVision XT 11.5 software, Noldus, Wageningen, Netherlands) recorded and analyzed all animal movements. The protocol for RTPP/CPP tests was taken from a reference (Tan et al., 2012). The paradigm consisted of three sessions over 5 days. On day 1, as a pretest session, mice freely explored the chamber for 15 min without light. We excluded mice showing a side preference higher than 35%. Days 2–4 were conditioning sessions; mice were trained for 30 min with photostimulation given in a light paired chamber. The chamber that was light paired was randomly assigned to each mouse in a counterbalanced manner. Photostimulation was triggered whenever mice entered the light-paired chamber, through a transistor–transistor logic (TTL) signal using a mini I/O box with EthoVision XT. To avoid overheating of the brain, lasers were turned off if mice stayed in the light-paired chamber for longer than 30 s. If mice continued to stay in the light-paired chamber, 1 min after the light pulse was turned off, the laser was turned on again. On day 5 as a posttest session, 24 h after the conditioning session, mice explored the chambers for 15 min without light.

Time in stim chamber (%) was calculated as (Time spent in the light-paired chamber)/(Time spent in either chamber) × 100.

$$\mathrm{Time in stim chamber}\;(\%)=\frac{\left(T_{\mathrm{light}-\mathrm{paired chamber}}\right)}{\left(T_{\mathrm{light}-\mathrm{paired chamber}}+T_{\mathrm{light}-\mathrm{unpaired chamber}}\right)}\times 100$$

### Histology

Mice were anesthetized and perfused with heparin sodium salt in phosphate-buffered saline (PBS) and then 4% formaldehyde in PBS. The brains were fixed overnight in 4% formaldehyde solution. After postfixation, the brains were sectioned (60 μm thickness) in a vibratome (Leica VT1200S, Leica, Wetzlar, Germany). Brain sections were mounted with Vectashield Hardset antifade mounting medium with 4′,6-diamidino-2-phenylindole (DAPI) (Vector Laboratories, Burlingame, CA, United States). Brain sections were imaged under a A1 HD25 high-resolution confocal microscope (Nikon, Tokyo, Japan) and analyzed using NIS-Elements AR analysis software (Nikon, Tokyo, Japan).

### Whole-Cell Patch-Clamp Recordings

Mouse brain slices were prepared at least 3 weeks after injection of AAV2/9.EF1α.DIO.hChR2 (H134R).mCherry into the MPA and AAV2/5.EF1α.DIO.eYFP into the vPAG. Whole-cell patch-clamp recordings were taken from the vPAG of 9–11-week-old mice. Mice were anesthetized with isoflurane and transcardially perfused with a cutting solution (220 mM sucrose, 26 mM NaHCO_3_, 2.5 mM KCl, 1 mM NaH_2_PO_4_, 5 mM MgCl2, 1 mM CaCl2, 10 mM glucose; pH 7.3–7.35). The mice were then decapitated, and the entire brain was removed and immediately submerged in ice-cold carbogen-saturated cutting solution. Then, 300-μm coronal sections were cut from the vPAG with a Leica VT1200S vibratome and incubated in oxygenated storage solution (123 mM NaCl, 26 mM NaHCO_3_, 2.8 mM KCl, 1.25 mM NaH_2_PO_4_, 1.2 mM MgSO_4_, 2.5 mM CaCl_2_, 10 mM glucose; pH 7.3–7.35) at 34°C for at least 1 h before recording. Slices were transferred to the recording chamber and allowed to equilibrate for 10 min before recording. Recordings were made in the presence of a recording solution (126 mM NaCl, 26 mM NaHCO_3_, 2.8 mM KCl, 1.25 mM NaH_2_PO_4_, 1.2 mM MgSO_4_, 2.5 mM CaCl_2_, 5 mM glucose; pH 7.3–7.35). The pipette solution for voltage-clamp, whole-cell recordings consisted of 120 mM potassium gluconate, 10 mM KCl, 10 mM 4-(2-hydroxyethyl)-1-piperazineethanesulfonic acid (HEPES), 5 mM ethylene glycol tetraacetic acid (EGTA), 1 mM CaCl_2_, 1 mM MgCl_2_, 2 mM MgATP; pH 7.29. Infrared differential interference contrast imaging was used to obtain the whole-cell recording (Nikon Eclipse FN-S2N equipped with a fixed stage and a QImaging optiMOS sCMOS camera). Electrophysiological signals were recorded using an Axopatch 700B amplifier (Molecular Devices, San Jose, CA, United States), low-pass filtered at 2–5 kHz and analyzed offline on a PC with Clampfit (Molecular Devices, San Jose, CA, United States). Recording electrodes had resistances of 2–6 MΩ when filled with the potassium gluconate internal solutions.

Photostimulation was delivered through an OptoPatcher (A-M Systems, Sequim, WA, United States), connected to a laser source (473 nm; Shanghai Lasers, Shanghai, China), through a patch cord with an NA of 4.8. Light intensity at the end of the optic fiber was measured as 0.4 mW. CRACM experiments (Petreanu et al., 2007) were conducted in voltage-clamp mode at −60 and −10 mV to detect excitatory and inhibitory postsynaptic currents, respectively. Three single light pulses (10 ms) were delivered 1 s apart by triggering a pulse generator with pClamp software.

### Statistics

No statistical analyses were performed to predetermine sample sizes. The sample sizes used were similar to those used in many previous studies (Kim et al., 2017; Park et al., 2018). All data analyses were performed using SigmaPlot (12.0; Systat Software). For parameters that followed a normal distribution (Shapiro–Wilk test, *p* > 0.05), differences between two groups were analyzed with the Student’s *t*-test, and comparisons of three or more groups were performed with the analysis of variance (ANOVA). The Wilcoxon signed-rank test, Mann–Whitney *U* test, and the signed-rank test were used for data that were not normally distributed. All statistical test were two-sided, and *p* < 0.05 were considered statistically significant.

## Results

### MPA^vgat^ Neurons Induce Exploration Without Reinforcement

**FIGURE 1:**
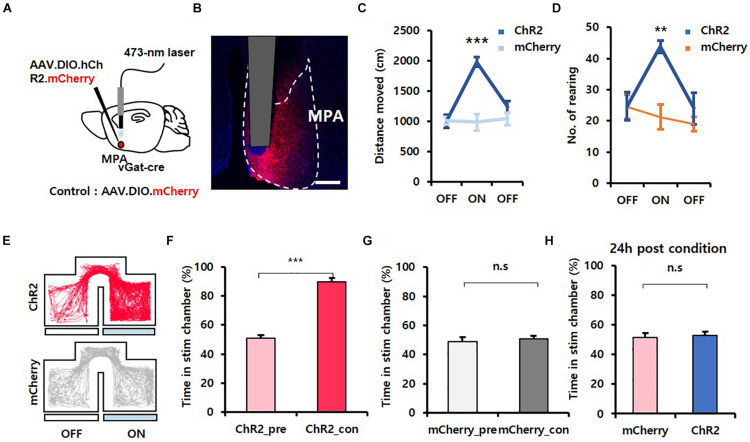
Activation of MPA^vgat^ neurons induces exploration but does not encode value. **(A)** Photoactivation of MPA^vgat^ neurons. Viruses were injected into the MPA, and fiber-optic cannulas were implanted above the injection site. **(B)** Virally mediated expression of ChR2 in MPA neurons of *vgat*-ires-*Cre* mice. Scale bar, 250 μm. **(C)** Photostimulation of MPA^vgat^ neuron increases movement in ChR2-expressing mice (*n* = 6; *n* = 3 males; *n* = 3 females) compared to mCherry control mice (*n* = 4; *n* = 1 male; *n* = 3 females); ***p < 0.001. Error bars represent SD. **(D)** Number of rearing increases during photostimulation of ChR2-expressing mice (*n* = 6; *n* = 3 males; *n* = 3 females) compared to control mice (*n* = 4; *n* = 1 male; *n* = 3 females); **p < 0.01. Error bars represent SD. **(E)** Representative movement trace of ChR2-expressing (red) mice and mCherry control (gray) mice during RTPP. **(F)** ChR2-expressing (*n* = 6; *n* = 3 males; *n* = 3 females) mice show real-time preference toward the stim chamber; ***p < 0.001. Error bars represent SD. **(G)** mCherry control (*n* = 4; *n* = 1 male; *n* = 3 females) do not show real-time place preference toward the stim chamber; *p* = 0.36. Error bars represent SD. **(H)** Conditioned place preference test 24 h after photostimulation with one chamber. mCherry control (*n* = 4; *n* = 1 male; *n* = 3 females) and ChR2-expressing (*n* = 6; *n* = 3 males; *n* = 3 females) mice do not have preference toward the stim chamber; *p* = 0.753. Error bars represent SD.

To investigate if GABAergic neurons in the MPA were associated with exploration behavior, we first performed optogenetic experiments (Zhang et al., 2006). We unilaterally injected adeno-associated virus (AAV) particles containing a Cre-dependent, channelrhodopsin (ChR2) virus (AAV.EF1α.DIO.hChR2.mCherry) into the MPA of *vgat*-ires-*Cre* mice and implanted fiber-optic cannulas over the vPAG. AAV.EF1α.DIO.mCherry virus was injected as a control (Figure 1A). Histology confirmed expression of the viruses in MPA neurons (Figure 1B). We conducted behavioral experiments 4 weeks after surgery. Blue illumination (473 nm, 20 Hz, 5 ms, 3 mW, pulse) was delivered through the implanted optic fiber to activate the soma of MPA^vgat^ neurons. In an open field test (OFT), an exploratory test (Brown et al., 1999), the locomotion of ChR2-expressing mice [mean ± standard deviation (SD); OFF, 1,008.16 ± 108.83; ON, 1,982.74 ± 79.03; OFF, 1,239.64 ± 98.66] showed a statistically significant increase compared to control mice (OFF, 1,012.89 ± 60.93; ON, 989.63 ± 139.43; OFF, 1,045.45 ± 101.37) {Figure 1C; time [*F*_(__2, 16)_ = 23.972, *p* < 0.001]; virus [*F*_(__1, 8)_ = 10.536, *p* = 0.012]; interaction [*F*_(__2, 16)_ = 27.724, *p* < 0.001]; two-way repeated measures ANOVA}. ChR2 mice (OFF, 24.67 ± 4.47; ON, 43.83 ± 2.04; OFF, 24.00 ± 5.04) also showed significantly more rearing than control mice (OFF, 24.5 ± 3.93; ON, 21.25 ± 3.92; OFF, 19.0 ± 2.27), a type of exploratory behavior, during the home cage test {Figure 1D; time [*F*_(__2, 16)_ = 6.208, *p* = 0.010]; virus [*F*_(__1, 8)_ = 4.421, *p* = 0.069]; interaction [*F*_(__2, 16)_ = 6.654, *p* = 0.008]; two-way repeated measures ANOVA}. Since exploration by mice is characterized by alternating bouts of rapid movement, progression, and lingering, we further analyzed locomotive patterns shown by each group of mice by log-transforming speeds of mouse trajectories and separating them into three clusters (slow, medium, fast) using the Expectation–Maximization algorithm (Drai et al., 2001) (Supplementary Figures 1A,B). Photostimulation resulted in a rightward shift in the mean of each cluster (ChR2 mice: slow, 0.22 ± 0.05; medium, 0.81 ± 0.02; fast, 1.40 ± 0.02; mCherry mice: slow, −0.12 ± 0.03; medium, 0.51 ± 0.05; fast, 1.20 ± 0.06) {Supplementary Figure 1C; cluster [*F*_(__2, 16)_ = 1,481.329, *p* < 0.001]; virus [*F*_(__1, 8)_ = 32.143, *p* < 0.001]; interaction [*F*_(__2, 16)_ = 4.234, *p* = 0.033]; two-way repeated measures ANOVA; Holm–Sidak *post-hoc* comparison *p* = 0.05}, representing an overall increase in locomotive speed but without changing the variance (ChR2 mice: slow, 0.31 ± 0.02; medium, 0.12 ± 0.00; fast, 0.03 ± 0.00; mCherry mice: slow, 0.30 ± 0.02; medium, 0.14 ± 0.01; fast, 0.04 ± 0.01) and weighting of each cluster (ChR2 mice: slow, 0.22 ± 0.05; medium, 0.56 ± 0.05; fast, 0.22 ± 0.01; mCherry mice: slow, 0.21 ± 0.02; medium, 0.58 ± 0.03; fast, 0.20 ± 0.01) {Supplementary Figures 1D,E, cluster [*F*_(__2, 16)_ = 257.533, *p* < 0.001]; virus [*F*_(__1, 8)_ = 0.702, *p* = 0.427]; interaction [*F*_(__2, 16)_ = 0.304, *p* = 0.742]; two-way repeated measures ANOVA; no statistically significant differences in Holm–Sidak *post-hoc* comparisons; 1E, cluster [*F*_(__2, 16)_ = 40.432, *p* < 0.001]; virus [*F*_(__1, 8)_ = +inf, *p* < 0.001]; interaction [*F*_(__2, 16)_ = 0.0874, *p* = 0.917]; two-way repeated measures ANOVA; no statistically significant differences in Holm–Sidak *post-hoc* comparisons}, thus showing that photostimulation does not alter the proportion of time spent during rapid movement, progression, or lingering. These results suggest that photostimulation of MPA^vgat^ neurons maintains the pattern of locomotion seen during explorative behavior. During the attached object test, the frequency in the object zone for ChR2-expressing mice (OFF, 7 ± 1.95; ON, 12 ± 2.16; OFF, 6.67 ± 1.71) increased during ON sessions when compared to control mice (OFF, 4 ± 0.41; ON, 4.25 ± 0.48; OFF, 1.75 ± 0.75) {Supplementary Figures 2A,B; time [*F*_(__2, 16)_ = 9.309, *p* = 0.002]; virus [*F*_(__1, 8)_ = 5.471, *p* = 0.047]; interaction [*F*_(__2, 16)_ = 3.337, *p* = 0.061]; two-way repeated measures ANOVA; Holm–Sidak *post-hoc* comparison *p* < 0.01}. Extrapolating with linear regression, we found that the increase in frequency in the object zone of ChR2-expressing mice was higher compared the mCherry-injected mice, even when considering their increased speed (Supplementary Figure 2C).

To test whether artificial activation of MPA^vgat^ neurons induced exploration and could be reinforcing, we conducted RTPP and CPP tests (Tan et al., 2012). An RTPP/CPP test box containing two chambers with distinct wall drawings and a corridor separating them was used. Mice freely explored the two chambers for 30 min/day, while receiving photostimulation of MPA^vgat^ neurons in one of the chambers, over 3 days. During pre- and posttest sessions, mouse freely explored the test box without photostimulation for 15 min. We found that the average time in the stimulation chamber during the conditioning session increased significantly when compared to the pretest session in ChR2 mice (ChR2_pre, 50.87 ± 2.10; ChR2_con, 89.89 ± 2.28) [Figures 1E,F; 1F, *t*(5) = 11.607, *p* < 0.001, paired *t*-test]. However, mCherry control mice showed no differences (mCherry_pre, 48.98 ± 3.10; mCherry_con, 50.69 ± 2.09) [Figure 1G; *t*(3) = 1.074, *p* = 0.361, paired *t-*test]. Interestingly, during the posttest session, 24 h postconditioning, ChR2-expressing mice (53.00 ± 2.46) and control mice (51.73 ± 3.09) showed no difference in time spent in the stimulation chamber [Figure 1H; *t*(8) = 0.325, *p* = 0.753, unpaired *t*-test]. To validate if activation of MPA^vgat^ neurons has a reinforcement effect, we analyzed the RTPP data across 5-min time windows. On conditioning day 1, ChR2-expressing mice did not show any progressive increases in the time spent in the stim chamber {Supplementary Figures 2B, 3A,B, C1_Time[*F*_(__5, 40)_ = 2.670, *p* = 0.036]; C2_Virus[*F*_(__1, 8)_ = 121.086, *p* < 0.001]; C_Interaction [*F*_(__5, 40)_ = 2.171, *p* = 0.077]; C2_Time [*F*_(__5, 40)_ = 8.148, *p* < 0.001]; C2_Virus [*F*_(__1, 8)_ = 67.964, *p* < 0.001]; C2_Interaction [*F*_(__5, 40)_ = 3.307, *p* = 0.014]; C3_Time [*F*_(__5, 40)_ = 3.005, *p* = 0.021]; C3_Virus [*F*_(__1, 8)_ = 104.320, *p* < 0.001]; C3_Interaction [*F*_(__5, 40)_ = 1.765, *p* = 0.142]; two-way repeated measures ANOVA; no statistically significant differences in Holm–Sidak post hoc comparisons}. The average speed in the stim chamber was significantly higher in ChR2-expressing mice (6.52 ± 0.15) than in control mice (4.78 ± 0.23) [Supplementary Figure 4A; *t*(8) = 7.099, *p* < 0.001; unpaired *t*-test]. Based on this CPP result, the observed preference in the stimulation chamber during the RTPP test can be explained due to an increase in exploration. Together, these results suggest that GABAergic neurons in the MPA can induce exploration behavior but fail to condition mice.

### MPA^vgat^ Neurons Send Inhibitory Output to vPAG^vgat^ Neurons

**FIGURE 2:**
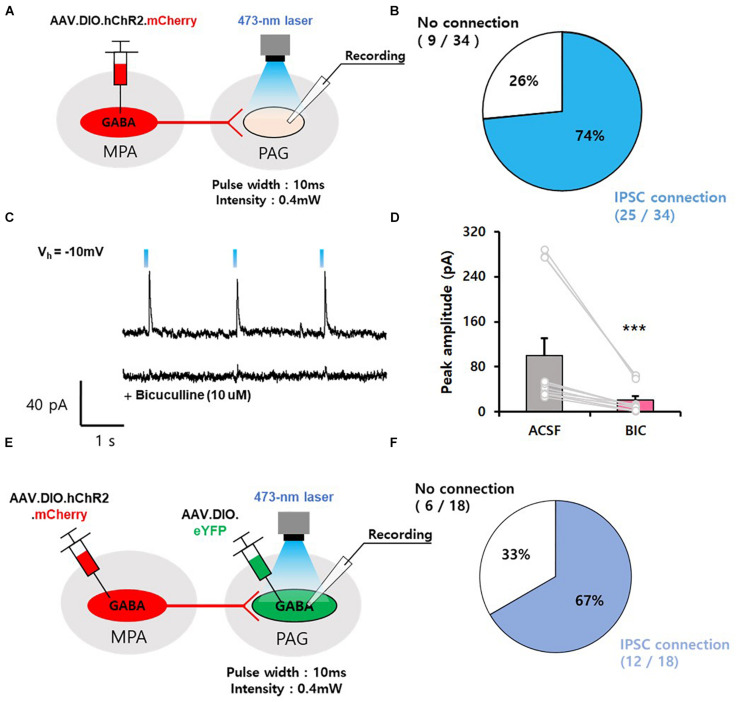
GABAergic neurons in the MPA send inhibitory input to GABAergic neurons in the vPAG. **(A)** Mapping connections from MPA^vgat^ neurons onto vPAG neurons using ChR2-assisted circuit mapping (CRACM). **(B)** Percentage of IPSCs evoked in postsynaptic vPAG neurons exposed to photostimulation. (IPSC connection, *n* = 2 5 cells; no connection, *n* = 9 cells). **(C)** Top, representative IPSC traces of vPAG neurons. Bottom, trace of single photostimulation, resulting in an IPSC, which was abolished by bicuculline (10 μM). **(D)** Photostimulation of MPA^vgat^ axon termini in the vPAG induces IPSCs that are abolished by pharmacological blockade of GABA_*A*_ receptors with bicuculline (*n* = 12); ***p < 0.001. Error bars represent SD. **(E)** Schematic of experiment, injection of AAV.DIO.hChR2.mCherry into the MPA and injection of AAV.DIO.eYFP into the vPAG of *vgat*-ires-*Cre* mice, followed by voltage clamp recordings of eYFP-positive cells in the vPAG. **(F)** Percentage of IPSCs evoked in postsynaptic vPAG^vgat^ neurons exposed to photostimulation. (IPSC connection, *n* = 12 cells; no connection, *n* = 6 cells).

To identify the functional connectivity of GABAergic neurons in the MPA, we performed whole-cell patch-clamp recordings. We injected AAV.EF1α.DIO.hChR2.mCherry into the MPA of *vgat*-ires-*Cre* mice and recorded cells in the vPAG (Figure 2A). We found that photoactivation of axonal termini of MPA^vgat^ neurons evoked inhibitory postsynaptic currents (IPSCs) in most (74%, 25 of 34) vPAG neurons, while only a subset (26%, 9 of 34) showed no connections (Figure 2B). The light-induced IPSCs in vPAG neurons were abolished in the presence of bicuculline (10 μM), an antagonist of GABA_*A*_ receptors (ACSF, 99.62 ± 31.38; BIC, 20.62 ± 7.34) (Figures 2C,D; 2D, Z-statistic = −3.059, *p* < 0.001; paired *t*-test) (Johnston et al., 1972). To investigate if GABAergic vPAG neurons receive input from MPA^vgat^ neurons, we injected AAV.EF1α.DIO.hChR2.mCherry into the MPA and AAV.EF1α.DIO.eYFP into the vPAG in *vgat*-ires-*Cre*. We recorded eYFP-expressing cells in the vPAG (Figure 2E). We found that MPA^vgat^ neurons send inhibitory input to vPAG^vgat^ neurons (67%, 12 of 18) (Figure 2F).

### MPA^vgat^–vPAG Projections Induce Exploration Behavior Without Reinforcement

**FIGURE 3:**
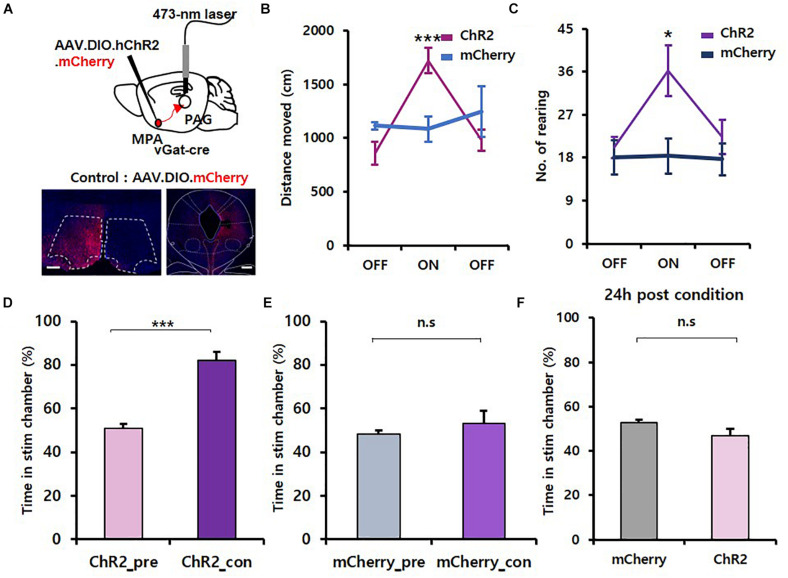
Activation of the MPA^vgat^–vPAG circuit induces exploration without encoding value. **(A)** Top, MPA–vPAG circuit photostimulation. Viruses were injected into the MPA, and fiber-optic cannulas were implanted over the vPAG. Bottom, virally mediated expression of ChR2 in MPA neurons of *vgat*-ires-*Cre* mice and axon termini in the vPAG. Scale bar, 250 μm. **(B)** Photoactivation of MPA^vgat^ neuron axon termini causes increased movement in ChR2-expressing mice (*n* = 7; *n* = 4 males; *n* = 3 females) compared to mCherry control mice (*n* = 3; *n* = 1 male; *n* = 2 females); ***p < 0.001. Error bars represent SD. **(C)** Number of rearing significantly increases in ChR2-expressing mice (*n* = 7; *n* = 4 male; *n* = 3 females) compared to mCherry control mice (*n* = 3; *n* = 1 male; *n* = 2 females); *p < 0.05. Error bars represent SD. **(D)** ChR2-expressing (*n* = 7; *n* = 4 males; *n* = 3 females) mice show more time in the stimulation chamber during the conditioning session compared to the pretest session; ***p < 0.001. Error bars represent SD. **(E)** mCherry control (*n* = 3; *n* = 1 male; *n* = 2 females) do not show real-time preference toward the stim chamber; *p* = 0.562. Error bars represent SD. **(F)** Testing for conditioned place preference 24 h after the conditioning session. mCherry control (*n* = 3; *n* = 1 male; *n* = 2 females) and ChR2-expressing (*n* = 7; *n* = 4 males; *n* = 3 females) mice do not show preference toward the stim chamber; *p* = 0.270. Error bars represent SD.

To activate GABAergic neurons in the MPA projecting to the vPAG, we injected AAV.EF1α.DIO.hChR2.mCherry virus unilaterally into the MPA of *vgat*-ires-*Cre* mice and implanted fiber-optic cannulas over the vPAG. We injected AAV.EF1α.DIO.mCherry virus into *vgat*-ires-*Cre* mice as a control (Figure 3A). Photoactivation of MPA^vgat^–vPAG projections significantly increased the distance moved (ChR2 mice: OFF, 856.95 ± 104.24; ON, 1,720.63 ± 114.89; OFF, 979.94 ± 101.37; mCherry mice: OFF, 1,115.85 ± 34.32; ON, 1,083.44 ± 118.61; OFF, 1,248.69 ± 236.16) {Figure 3B; time [*F*_(__2, 16)_ = 7.894, *p* = 0.004]; virus [*F*_(__1, 8)_ = 0.0613, *p* = 0.811]; interaction [*F*_(__2, 16)_ = 11.787, *p* < 0.001]; two-way repeated measures ANOVA} and number of rearing in ChR2 mice (ChR2 mice: OFF, 20.14 ± 2.18; ON, 36.29 ± 5.29; OFF, 22.29 ± 3.58; mCherry mice: OFF, 18.00 ± 3.61; ON, 18.33 ± 3.76; OFF, 17.67 ± 3.38; Figure 3C; time [*F*_(__2, 16)_ = 6.371, *p* = 0.009]; virus [*F*_(__1, 8)_ = 1.997, *p* = 0.195]; interaction [*F*_(__2, 16)_ = 5.643, *p* = 0.014]; two-way repeated measures ANOVA}. Likewise, analysis of locomotive patterns showed that photostimulation of the MPA^vgat^–vPAG projection during the OFT resulted in a rightward shift of each cluster distribution (ChR2 mice: slow, 0.20 ± 0.05; medium, 0.72 ± 0.03; fast, 1.38 ± 0.03; mCherry mice: slow, −0.43 ± 0.34; medium, 0.39 ± 0.12; fast, 1.29 ± 0.01), without affecting the variance distribution (ChR2 mice: slow, 0.25 ± 0.02; medium, 0.13 ± 0.01; fast, 0.02 ± 0.00; mCherry mice: slow, 0.23 ± 0.07; medium, 0.18 ± 0.02; fast, 0.03 ± 0.01) or the overall weighting distribution (ChR2 mice: slow, 0.22 ± 0.04; medium, 0.56 ± 0.04; fast, 0.22 ± 0.01; mCherry mice: slow, 0.11 ± 0.06; medium, 0.73 ± 0.07; fast, 0.16 ± 0.03) of the cluster toward the overall {Supplementary Figures 1F–J, cluster [*F*_(__2, 16)_ = 157.775, *p* < 0.001]; virus [*F*_(__1, 8)_ = 12,542, *p* = 0.008]; interaction [*F*_(__2, 16)_ = 5.402, *p* = 0.016]; two-way repeated measures ANOVA; Holm–Sidak *post-hoc* comparison *p* = 0.05; 1I, cluster [*F*_(__2, 16)_ = 42.795, *p* < 0.001]; virus [*F*_(__1, 8)_ = 0.290, *p* = 0.605]; interaction [*F*_(__2, 16)_ = 1.067, *p* = 0.367]; two-way repeated measures ANOVA; no statistically significant differences in Holm–Sidak *post-hoc* comparisons; 1J, cluster [*F*_(__2, 16)_ = 49.587, *p* < 0.001]; virus [*F*_(__1, 8)_ = +inf, *p* < 0.001]; interaction [*F*_(__2, 16)_ = 3.984, *p* = 0.039]; two-way repeated measures ANOVA; no statistically significant differences in Holm–Sidak *post-hoc* comparisons, except medium; *p* = 0.013}. In the attached object test, the frequency in object zone shown by ChR2-expressing mice (OFF, 4.29 ± 1.06; ON, 6.43 ± 1.17; OFF, 4.14 ± 0.88) was not different from control mice (OFF, 3.67 ± 1.67; ON, 2.67 ± 0.67; OFF, 2.00 ± 0.58) {Supplementary Figure 2D; time [*F*_(__2, 16)_ = 1.137, *p* = 0.345]; virus [*F*_(__1, 8)_ = 2.540, *p* = 0.150]; interaction [*F*_(__2, 16)_ = 1.267, *p* = 0.308]; two-way repeated measures ANOVA}, although we found the correlation between frequency in object zone and velocity to be steeper for ChR2-injected mice (Supplementary Figure 2E).

To test whether artificial activation of MPA^vgat^–vPAG projections can drive reinforcement, we conducted RTPP/CPP tests. We found that photoactivation of MPA^vgat^–vPAG projections (ChR2_pre, 50.76 ± 2.42; ChR2_con, 81.91 ± 4.07) induced preference in real-time in ChR2 mice [Figure 3D; *t*(6) = 6.885, *p* < 0.001, paired *t*-test], whereas control mice (mCherry_pre, 48.36 ± 1.55; mCherry_con, 53.11 ± 5.75) showed no changes [Figure 3E; *t*(2) = 0.689, *p* = 0.562, paired *t*-test]. Twenty-four hours after the conditioning session, neither ChR2-expressing mice (46.73 ± 3.07) nor control mice (52.59 ± 1.47) showed preference to the stimulation chamber [Figure 3F; *t*(8) = 1.186, *p* = 0.270, unpaired *t*-test]. To validate if activation of the MPA^vgat^–svPAG projection has a reinforcement effect, we analyzed the RTPP data across 5-min time windows. On conditioning day 1, ChR2-expressing mice show no progressive increase in the time spent in the stim chamber {Supplementary Figure 2C, 3C, C1_Time [*F*_(__5, 40)_ = 2.266, *p* = 0.066]; C1_Virus [*F*_(__1, 8)_ = 13.066, *p* = 0.007]; C1_Interaction [*F*_(__5, 40)_ = 1.463, *p* = 0.224]; C2_Time [*F*_(__5, 40)_ = 1.782, *p* = 0.139]; C2_Virus [*F*_(__1, 8)_ = 22.887, *p* = 0.001]; C2_Interaction [*F*_(__5, 40)_ = 0.634, *p* = 0.675]; C3_Time [*F*_(__5, 40)_ = 0.152, *p* = 0.978]; C3_Virus [*F*_(__1, 8)_ = 9.834, *p* = 0.014]; C3_Interaction [*F*_(__5, 40)_ = 1.154, *p* = 0.349]; two-way repeated measures ANOVA; no statistically significant differences in Holm–Sidak *post-hoc* comparisons}. The average speed in stim chamber did not show any difference in ChR2-expressing mice (5.57 ± 0.13) than control mice (5.00 ± 0.46) (Supplementary Figure 4A; U = 4.000, *p* = 0.262, Mann–Whitney *U* test). To test whether only activation of MPA^vgat^ neuron increases the speed of ChR2-expressing mice in the stimulation chamber, we compared the average speed of the first and second entry into the stimulation chamber. Activation of MPA^vgat^ ChR2-expressing mice (8.73 ± 0.46) caused an increase in average speed in comparison to activation of MPA^vgat^–vPAG ChR2-expressing mice (7.27 ± 0.41) [Supplementary Figures 3C, 4B,C, *t*(11) = 2.404, *p* = 0.035, unpaired *t*-test]. Thus, these results show that activation of the MPA GABAergic projection to the vPAG induces exploration behavior but fails to condition mice.

### Inhibition of the MPA^vgat^–vPAG Circuit

**FIGURE 4:**
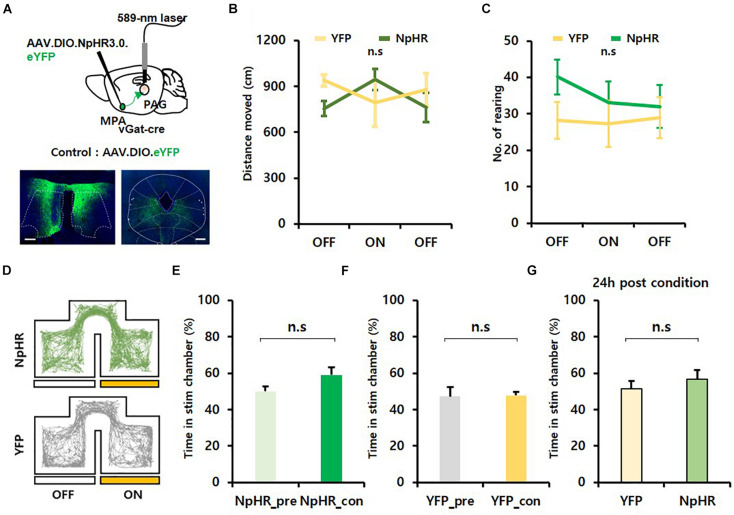
Photoinhibition of GABAergic neurons in the MPA projecting to the vPAG does not alter exploration and does not encode value. **(A)** Top, MPA–vPAG circuit photoinhibition induced by a 589-nm laser. AAV.DIO.eNpHR3.0.eYFP was bilaterally injected into the MPA, and dual-fiber optic cannulas were implanted over the vPAG. Bottom, virally mediated expression of NpHR in the MPA of *vgat*-ires-*Cre* mice and axon termini in the vPAG. Scale bar, 250 μm. **(B)** NpHR-expressing mice (*n* = 7; *n* = 5 males; *n* = 2 females) have no significant increase in distance moved compared to eYFP control mice (*n* = 4; *n* = 1 male; *n* = 3 females); OFF_pre, *p* = 0.169; ON, *p* = 0.245; OFF_post, *p* = 0.380. Error bars represent SD. **(C)** No difference in the number of rearing between NpHR-expressing mice (*n* = 7; *n* = 5 males; *n* = 2 females) and eYFP-expressing mice (*n* = 4; *n* = 1 male; *n* = 3 females); *p* = 0.431. Error bars represent SD. **(D**) Representative movement trace of NpHR-expressing (green) mice and eYFP control mice (gray) during RTPP. **(E)** NpHR-expressing (*n* = 7; *n* = 5 males; *n* = 2 females) mice show no significant difference in time spent in the stim chamber during RTPP; *p* = 0.098. Error bars represent SD. **(F)** eYFP control (*n* = 4; *n* = 1 male; *n* = 3 females) have no difference in time spent in the stim chamber during RTPP; *p* = 0.923. Error bars represent SD. **(G)** Twenty-four hours after the conditioning session. eYFP control (*n* = 4; *n* = 1 male; *n* = 3 females) and NpHR-expressing (*n* = 7; *n* = 5 males; *n* = 2 females) mice do not have preference toward the stim chamber; *p* = 0.488. Error bars represent SD.

To investigate whether photoinhibition of the MPA^vgat^–vPAG projection can reduce exploration, we injected AAV.EF1α.DIO.eNpHR3.0.eYFP virus bilaterally into the MPA of vgat-ires-*Cre* and implanted dual fiber-optic cannulas over the vPAG. We injected AAV.EF1α.DIO.eYFP virus into the MPA of *vgat*-ires-*Cre* mice as a control. Mice received continuous yellow light (589 nm, 20 mW) to inhibit MPA^vgat^ axon terminals in the vPAG (Figure 4A). Photoinhibition of MPA^vgat^–vPAG projections did not significantly change the distance moved (NpHR mice: OFF, 755.17 ± 50.52; ON, 946.18 ± 68.81; OFF, 762.15 ± 96.75; YFP mice: OFF, 938.95 ± 38.80; ON, 791.74 ± 152.98; OFF, 877.68 ± 107.78) {Figure 4B; time [*F*_(__2, 18)_ = 0.268, *p* = 0.768]; virus [*F*_(__1, 9)_ = 0.221, *p* = 0.650]; interaction [*F*_(__2, 18)_ = 3.555, *p* = 0.05]; OFF_pre, *p* = 0.169; ON, *p* = 0.245; OFF_post, *p* = 0.380; two-way repeated measures ANOVA} or number of rearing in NpHR or control mice {NpHR mice: OFF, 40.14 ± 4.76; ON, 33.00 ± 5.83; OFF, 32.00 ± 5.93; YFP mice: OFF, 28.25 ± 5.02; ON, 27.25 ± 6.42; OFF, 29.00 ± 5.61} {Figure 4C; time [*F*_(__2, 18)_ = 0.863, *p* = 0.439]; virus [*F*_(__1, 9)_ = 0.822, *p* = 0.388]; interaction [*F*_(__2, 18)_ = 0.883, *p* = 0.431]; two-way repeated measures ANOVA}. To verify whether artificial inhibition of MPA^vgat^–vPAG projections inhibit exploration or reinforcement, we performed RTPP and CPP. We found that photoinhibition of MPA^vgat^–vPAG projections (NpHR_pre, 50.01 ± 2.93; NpHR_con, 59.06 ± 4.35) did not induce preference in real time, in both NpHR and YFP control mice (YFP_pre, 47.26 ± 5.04; YFP_con, 47.82 ± 2.06) [Figures 4D–F, *t*(6) = 1.958, *p* = 0.098; 4F, *t*(3) = 0.105, *p* = 0.923; paired *t*-test]. Twenty-four hours after conditioning, NpHR-expressing mice (56.76 ± 4.94) and control mice (51.45 ± 4.16) showed no preference toward the stimulation chamber [Figure 4G; *t*(9) = 0.723, *p* = 0.488; unpaired *t*-test]. These findings suggest that inhibition of MPA^vgat^–vPAG projections do not cause preference and do not decrease movement.

## Discussion

In the present study, we reveal that vPAG projecting MPA^vgat^ neurons induce exploration and that these vgat neurons in the MPA send inhibitory input to vgat neurons of the vPAG. We found that activation of MPA^vgat^ neurons increases locomotion and time spent in the stimulation chamber during RTPP but do not show any preference during CPP. We interpreted this increased time spent in the simulation chamber during real-time tests as not being reinforcing but as increasing exploration. We recapitulated this result when activating the MPA^vgat^–vPAG circuit. However, inhibition of the MPA^vgat^–vPAG circuit did not decrease exploration behavior.

### Activation of MPA GABAergic Neurons Is Not Reinforcing

A previous study showed that vgat neurons, a subset of GABAergic neurons, in the MPA encode positive value (Zhang et al., 2021). Indeed, we recapitulated the results from the RTPP and open field tests in this study. However, our study showed that activation of MPA^vgat^ neurons and MPA^vgat^–vPAG projections did not induce any difference in time spent in a stimulation chamber 24 h after a conditioning session (Figures 1H, 3F), suggesting that the increased preference seen during RTPP was not due to a reinforcing effect. We found that there was no progressive increase in preference during RTPP tests, consistent with the notion that activation of MPA^vgat^ neurons does not have a reinforcing effect. One important caveat to note is that we cannot rule out the hypothesis that these effects may have been caused by contextual memory impairments induced by activation of MPA neurons. Taken together, we tentatively interpret these results to mean that MPA^vgat^ neurons promote exploration in a non-reinforcing manner, although further experiments will be needed to conclusively rule out a change in memory.

### Inhibition of MPA GABAergic Neurons Does Not Decrease Exploration

During photoinhibition of the MPA^vgat^–vPAG circuit, we found no decreases in either locomotion or time spent in the stimulation chamber during an RTPP test. Although these results may tentatively suggest that MPA^vgat^–vPAG is not necessary for exploration, it is important to note that our experiments did not include other stimuli during the test. Previous studies on the function of MPA neurons projecting to the vPAG have shown that these neurons respond to particular stimuli, such as CaMKIIα neurons responding to an object or prey, vglut2 neurons responding to stress, and galanin neurons responding to pups (Wu et al., 2014; Kohl et al., 2018; Park et al., 2018; Zhang et al., 2021). Thus, it is plausible that there will be certain stimuli or conditions that could cause activation in the MPA^vgat^–vPAG circuit that in turn facilitate exploration. The absence of such a stimulus would result in no difference in exploration when inhibiting this circuit, which may account for our results. Furthermore, the vPAG is likely to receive presynaptic input from other brain regions, which may have also contributed to the lack of any effect seen from inhibiting the MPA^vgat^–vPAG circuit. Further studies will be needed to reveal the detailed function of this circuit.

### The MPA Is Associated With Broad Exploration

Exploratory behavior can consist of orienting responses, locomotor responses, and investigatory responses, which refer to orientation of sensory organs, displacement of the whole body, and manipulation of objects in the environment, respectively (Hughes, 1997). It is thus possible that the MPA is more broadly involved with all types of exploratory behaviors, whereas the MPA–vPAG connection is restricted to only mediating the locomotory responses of exploration, without affecting investigatory responses (Supplementary Figure 2). This would explain why photoactivation of MPA–vPAG resulted in preference during RTPP and increased locomotion but no changes in object exploration. However, this locomotory response appears to occur in a non-reinforcing manner, since photostimulation of the MPA–vPAG projection could not condition mice during CPP. Notably, this increase in locomotion occurred independently of changes in locomotor speed, suggesting that it was not a direct motor effect (Supplementary Figure 3). Further analysis of locomotor patterns showed that alternations between lingering, progressing, and rapid movement, which are hallmarks of exploratory behavior (Drai et al., 2001), were maintained during photostimulation (Supplementary Figure 1). Since the medial septum is known to project to the MPA, which has been shown to mediate speed increases during locomotion (Zhang et al., 2018), it is plausible that direct activation of MPA neurons can result in changes in speed during photostimulation. On the other hand, such changes in speed may be dependent on specific projections of the MPA to downstream sites, rather than a generalized effect, which may account for why the MPA–vPAG projection did not show any changes in speed during photostimulation (Supplementary Figure 3). Overall, our results suggest that MPA^vgat^ neurons are involved in mediating exploration in a non-reinforcing manner, which is essential for organisms in familiarizing themselves with new environments.

## Data Availability Statement

The original contributions presented in the study are included in the article/Supplementary Material, further inquiries can be directed to the corresponding author/s.

## Ethics Statement

The animal study was reviewed and approved by the Korean Advanced Institute of Science and Technology (KAIST) Guidelines for the Care and Use of Laboratory Animals and were approved by the Institutional Animal Care and Use Committee (Protocol No. KA2020-63).

## Author Contributions

DK designed the study and coordinated the experiments. JR performed the behavioral, optogenetic, and histological experiments and analyzed the data. SP performed the electrophysiology experiments. All authors participated in writing the manuscript.

## Conflict of Interest

The authors declare that the research was conducted in the absence of any commercial or financial relationships that could be construed as a potential conflict of interest.

## Publisher’s Note

All claims expressed in this article are solely those of the authors and do not necessarily represent those of their affiliated organizations, or those of the publisher, the editors and the reviewers. Any product that may be evaluated in this article, or claim that may be made by its manufacturer, is not guaranteed or endorsed by the publisher.

## Supplementary Material

Supplementary Figure 1 The pattern of the density function for speeds was conserved. **(A)** The distributions were fitted with the EM algorithm. The density estimation for the distribution of the log speed for the ChR2 group (*n* = 6; *n* = 3 males; *n* = 3 females) during MPA^vgat^ neural photoactivation in the OFT. **(B)** The density estimation for the distribution of the log speed for the mCherry group (*n* = 4; *n* = 1 male; *n* = 3 females). **(C)** The mean of each distribution for slow, medium, and fast clusters in the OFT; slow: ^∗∗∗^*p* < 0.001; medium: ^∗∗∗^*p* < 0.001; fast: ^∗∗^*p* = 0.003. Error bars represent SD. **(D)** The variance of each distribution for slow, medium, and fast clusters in the OFT; slow: *p* = 0.964; medium: *p* = 0.330; fast: *p* = 0.541. Error bars represent SD. **(E)** The weight of each distribution for slow, medium, and fast clusters during the OFT; slow: *p* = 0.861; medium: *p* = 0.683; fast: *p* = 0.814. Error bars represent SD. **(F)** The density estimation for the distribution of the log speed for the MPA^vgat^-PAG ChR2-expressing group (*n* = 7; *n* = 4 males; *n* = 3 females) during photoactivation in OFT. **(G)** The density estimation for the distribution of the log speed for the mCherry control group (*n* = 3; *n* = 1 male; *n* = 2 female). **(H)** The mean of each distribution for slow, medium, and fast clusters during photostimulation of PAG-projecting MPA^vgat^ neurons in the OFT; slow: ^∗∗∗^*p* < 0.001; medium: ^∗^*p* = 0.025; fast: *p* = 0.534. Error bars represent SD. **(I)** The variance of each distribution for slow, medium, and fast clusters during photostimulation of PAG-projecting MPA^vgat^ neurons in the OFT; slow: *p* = 0.537; medium: *p* = 0.169; fast: *p* = 0.889. Error bars represent SD. **(J)** The weight of each distribution for slow, medium, and fast clusters during photostimulation of PAG-projecting MPA^vgat^ neurons in the OFT.

Supplementary Figure 2 MPA^vgat^ activation increases approaches toward an object while activating the MPA^vgat^-vPAG projection does not. **(A)** Design of attached object test. The green square represents the object zone. **(B)** Top, photoactivation of MPA^vgat^ neuron increases the frequency in the object zone in ChR2-expressing mice (*n* = 6; *n* = 3 males; *n* = 3 females) compared to mCherry control mice (*n* = 4; *n* = 1 male; *n* = 3 females); OFF_pre: *p* = 0.249; ON: ^∗∗^*p* < 0.01; OFF_post: *p* = 0.071. Error bars represent SD. Bottom, raw trace from a ChR2-expressing mice. **(C)** Correlations between velocity and the frequency in object zone during photoactivation of MPA^vgat^ neurons in ChR2-expressing mice (*n* = 6; *n* = 3 males; *n* = 3 females) and mCherry control mice (*n* = 4; *n* = 1 male; *n* = 3 females). **(D)** Top, photoactivation of the MPA^vgat^-vPAG projection does not increase the frequency in the object zone in ChR2-expressing mice (*n* = 7; *n* = 4 males; *n* = 3 females) compared to control mice (*n* = 3; *n* = 1 male; *n* = 2 females); *p* = 0.308. Error bars represent SD. Bottom, raw trace from a ChR2-expressing mice. **(E)** Correlations between velocity and the frequency in object zone during phtoactivation of MPA^vgat^-PAG projecting neurons in ChR2-expressing mice (*n* = 7; *n* = 4 males; *n* = 3 females) and control mice (*n* = 3; *n* = 1 male; *n* = 2 females).

Supplementary Figure 3 Photoactivation of MPA^vgat^ neurons and the MPA^vgat^-vPAG circuit did not induce a reinforcement effect. **(A)** Illustration of Real-time place preference/conditioned place preference tests. **(B)** MPA^vgat^ activation in ChR2-expressing mice (*n* = 6; *n* = 3 males; *n* = 3 females) does not progressively increase the time spent in the stim chamber, for every 5 min window during conditioning sessions. mCherry control mice (*n* = 4; *n* = 1 male; *n* = 3 females) do not show any preference. **(C)** Photoactivation of the MPA^vgat^-vPAG projection in ChR2-expressing mice (*n* = 7; *n* = 4 males; *n* = 3 females) does not progressively increase the time spent in the stim chamber, for every 5 min window during the conditioning session. mCherry control mice (*n* = 3; *n* = 1 male; *n* = 2 females) do not show any preference.

Supplementary Figure 4 MPA^vgat^ activation promotes increased speed while MPA^vgat^-vPAG does not. **(A)** Average speed in stim chamber. Photostimulation of MPA^vgat^ neurons increases speed in ChR2-expressing mice (*n* = 6; *n* = 3 males; *n* = 3 females) compared to mCherry control mice (*n* = 4; *n* = 1 male; *n* = 3 females); ^∗∗∗^*p <* 0.001. Photostimulation of the MPA^vgat^-vPAG projection shows no significant changes in ChR2-expressing mice (*n* = 7; *n* = 4 males; *n* = 3 females) versus control mice (*n* = 3; *n* = 1 male; *n* = 2 females); *p* = 0.262. Error bars represent SD. **(B)** Representative locomotion speed plot during an entry trial while stimulating MPA^vgat^ neurons (red) and MPA^vgat^-vPAG axon terminals (black) in the real-time place preference test. **(C)** MPA^vgat^ stimulated mice (*n* = 6; *n* = 3 males; *n* = 3 females) increase their average speed on the first and second entry in the stimulation chamber, when compared to MPA^vgat^-vPAG axon terminal stimulation (*n* = 7; *n* = 4 males; *n* = 3 females); ^∗^*p* < 0.05. Error bars represent SD.
