# Supplementary figures and images for: An Inhibitory Medial Preoptic Circuit Mediates Innate Exploration
Source: Front Neurosci. 2021 Aug 23;15:716147. doi: 10.3389/fnins.2021.716147 (PMC8419349; doi:10.3389/fnins.2021.716147)

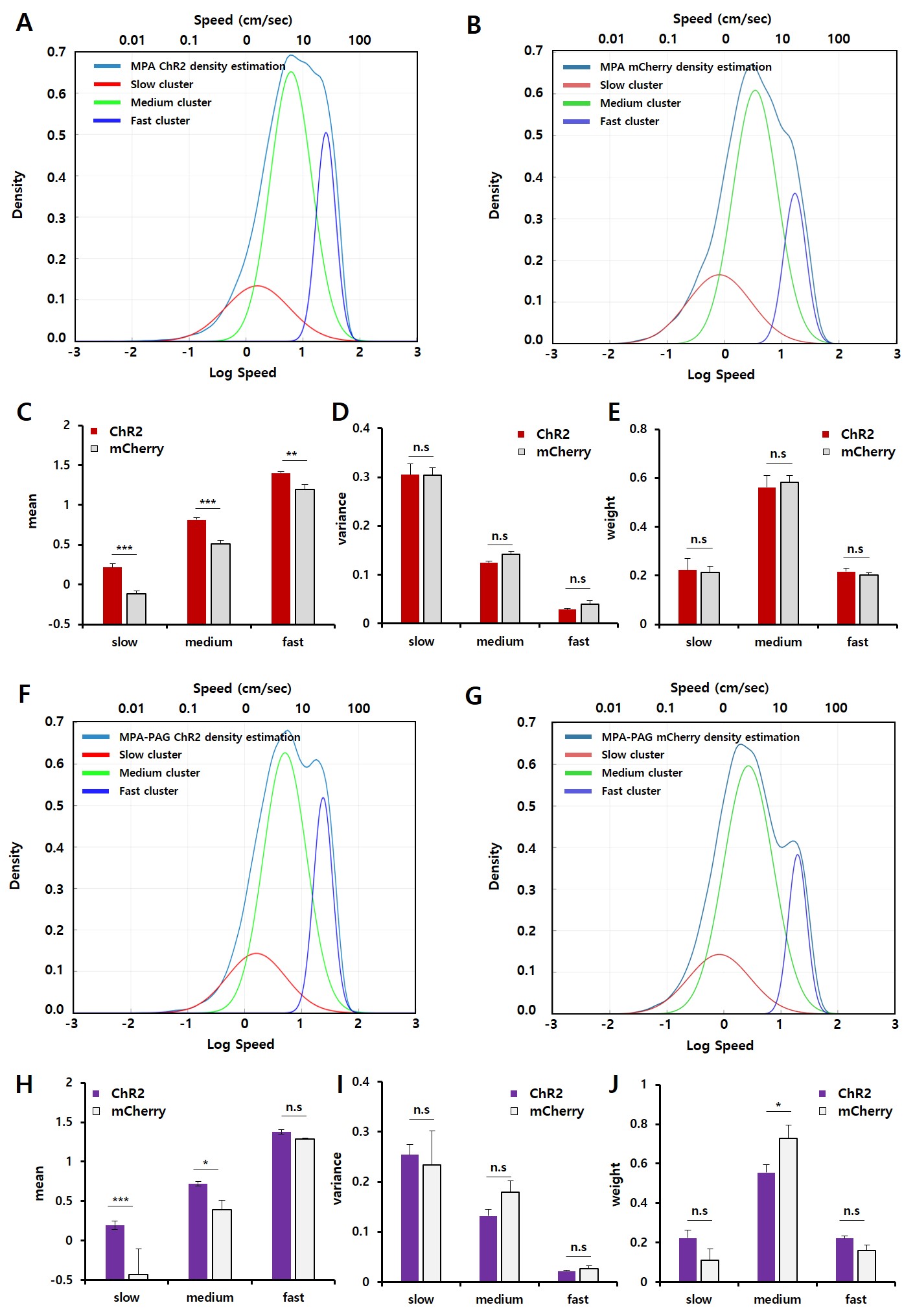

Supplement: Supplementary Figure 1 — The pattern of the density function for speeds was conserved. (A) The distributions were fitted with the EM algorithm. The density estimation for the distribution of the log speed for the ChR2 group (n = 6; n = 3 males; n = 3 females) during MPAvgat neural photoactivation in the OFT. (B) The density estimation for the distribution of the log speed for the mCherry group (n = 4; n = 1 male; n = 3 females). (C) The mean of each distribution for slow, medium, and fast clusters in the OFT; slow: ∗∗∗p < 0.001; medium: ∗∗∗p < 0.001; fast: ∗∗p = 0.003. Error bars represent SD. (D) The variance of each distribution for slow, medium, and fast clusters in the OFT; slow: p = 0.964; medium: p = 0.330; fast: p = 0.541. Error bars represent SD. (E) The weight of each distribution for slow, medium, and fast clusters during the OFT; slow: p = 0.861; medium: p = 0.683; fast: p = 0.814. Error bars represent SD. (F) The density estimation for the distribution of the log speed for the MPAvgat-PAG ChR2-expressing group (n = 7; n = 4 males; n = 3 females) during photoactivation in OFT. (G) The density estimation for the distribution of the log speed for the mCherry control group (n = 3; n = 1 male; n = 2 female). (H) The mean of each distribution for slow, medium, and fast clusters during photostimulation of PAG-projecting MPAvgat neurons in the OFT; slow: ∗∗∗p < 0.001; medium: ∗p = 0.025; fast: p = 0.534. Error bars represent SD. (I) The variance of each distribution for slow, medium, and fast clusters during photostimulation of PAG-projecting MPAvgat neurons in the OFT; slow: p = 0.537; medium: p = 0.169; fast: p = 0.889. Error bars represent SD. (J) The weight of each distribution for slow, medium, and fast clusters during photostimulation of PAG-projecting MPAvgat neurons in the OFT. [file Image_1.JPEG]

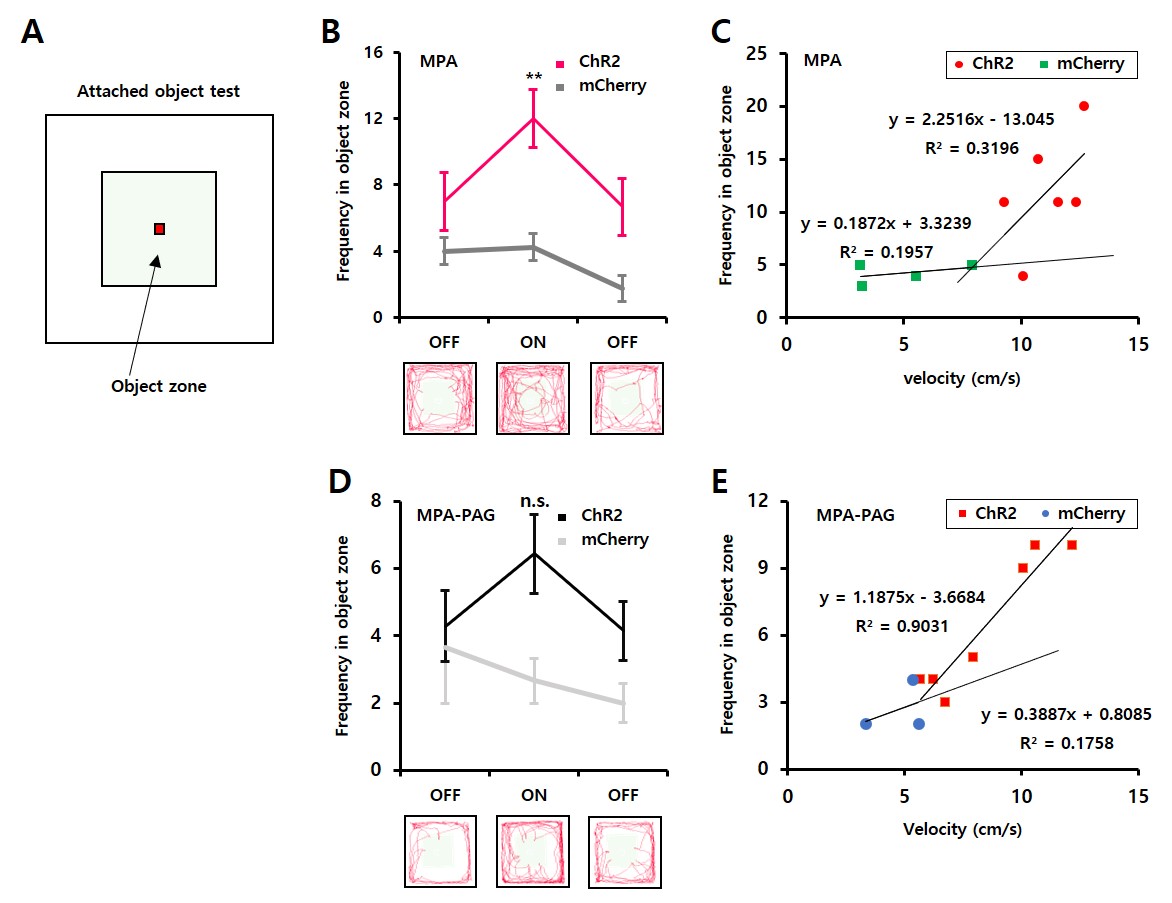

Supplement: Supplementary Figure 2 — MPAvgat activation increases approaches toward an object while activating the MPAvgat-vPAG projection does not. (A) Design of attached object test. The green square represents the object zone. (B) Top, photoactivation of MPAvgat neuron increases the frequency in the object zone in ChR2-expressing mice (n = 6; n = 3 males; n = 3 females) compared to mCherry control mice (n = 4; n = 1 male; n = 3 females); OFF_pre: p = 0.249; ON: ∗∗p < 0.01; OFF_post: p = 0.071. Error bars represent SD. Bottom, raw trace from a ChR2-expressing mice. (C) Correlations between velocity and the frequency in object zone during photoactivation of MPAvgat neurons in ChR2-expressing mice (n = 6; n = 3 males; n = 3 females) and mCherry control mice (n = 4; n = 1 male; n = 3 females). (D) Top, photoactivation of the MPAvgat-vPAG projection does not increase the frequency in the object zone in ChR2-expressing mice (n = 7; n = 4 males; n = 3 females) compared to control mice (n = 3; n = 1 male; n = 2 females); p = 0.308. Error bars represent SD. Bottom, raw trace from a ChR2-expressing mice. (E) Correlations between velocity and the frequency in object zone during phtoactivation of MPAvgat-PAG projecting neurons in ChR2-expressing mice (n = 7; n = 4 males; n = 3 females) and control mice (n = 3; n = 1 male; n = 2 females). [file Image_2.JPEG]

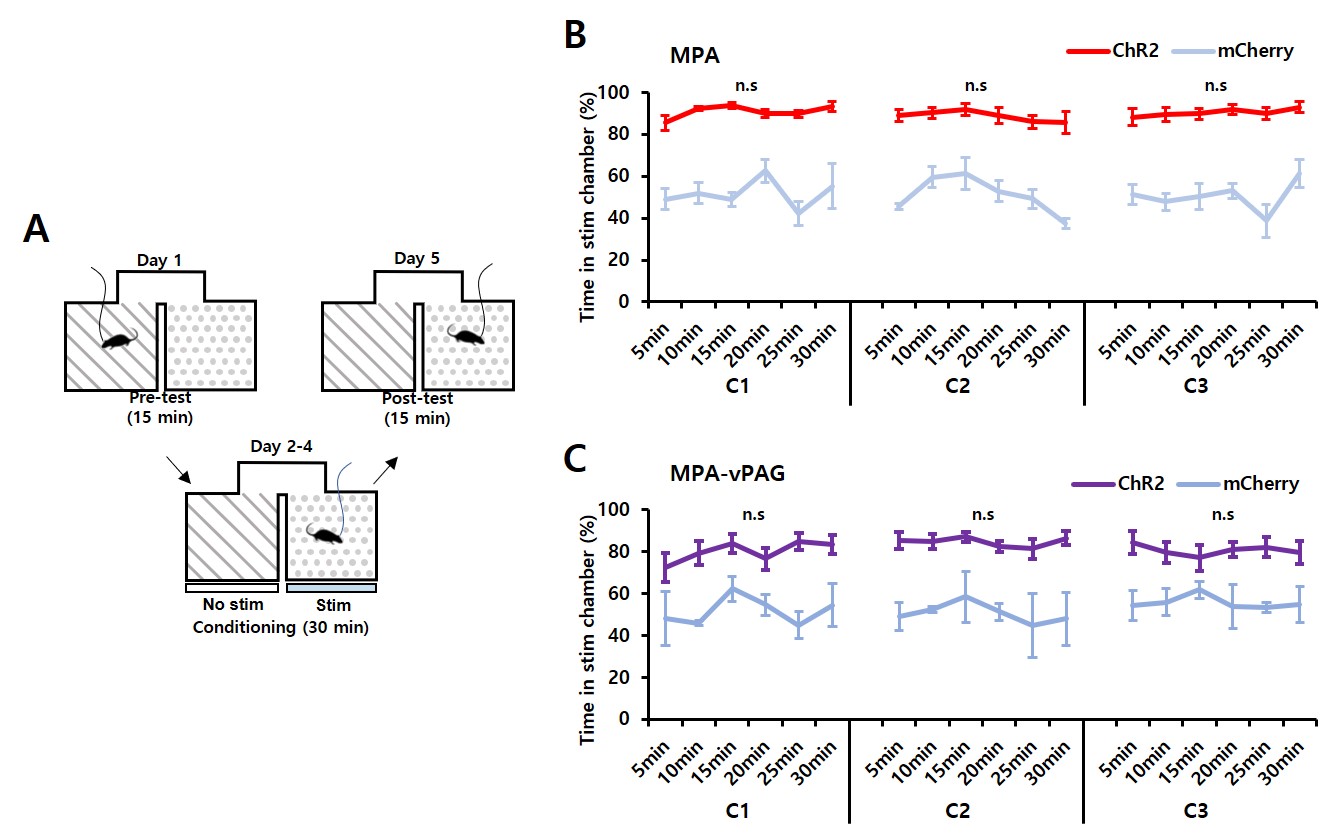

Supplement: Supplementary Figure 3 — Photoactivation of MPAvgat neurons and the MPAvgat-vPAG circuit did not induce a reinforcement effect. (A) Illustration of Real-time place preference/conditioned place preference tests. (B) MPAvgat activation in ChR2-expressing mice (n = 6; n = 3 males; n = 3 females) does not progressively increase the time spent in the stim chamber, for every 5 min window during conditioning sessions. mCherry control mice (n = 4; n = 1 male; n = 3 females) do not show any preference. (C) Photoactivation of the MPAvgat-vPAG projection in ChR2-expressing mice (n = 7; n = 4 males; n = 3 females) does not progressively increase the time spent in the stim chamber, for every 5 min window during the conditioning session. mCherry control mice (n = 3; n = 1 male; n = 2 females) do not show any preference. [file Image_3.JPEG]

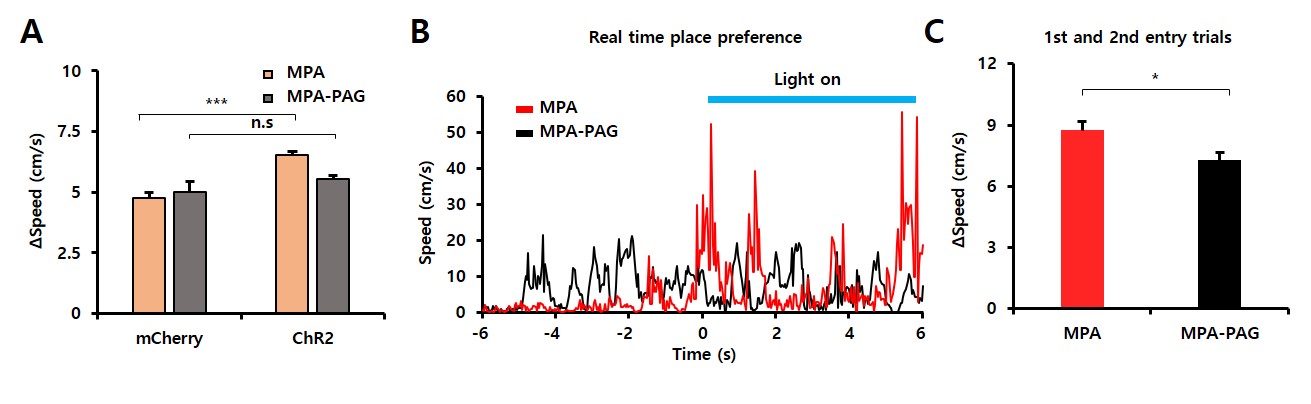

Supplement: Supplementary Figure 4 — MPAvgat activation promotes increased speed while MPAvgat-vPAG does not. (A) Average speed in stim chamber. Photostimulation of MPAvgat neurons increases speed in ChR2-expressing mice (n = 6; n = 3 males; n = 3 females) compared to mCherry control mice (n = 4; n = 1 male; n = 3 females); ∗∗∗p < 0.001. Photostimulation of the MPAvgat-vPAG projection shows no significant changes in ChR2-expressing mice (n = 7; n = 4 males; n = 3 females) versus control mice (n = 3; n = 1 male; n = 2 females); p = 0.262. Error bars represent SD. (B) Representative locomotion speed plot during an entry trial while stimulating MPAvgat neurons (red) and MPAvgat-vPAG axon terminals (black) in the real-time place preference test. (C) MPAvgat stimulated mice (n = 6; n = 3 males; n = 3 females) increase their average speed on the first and second entry in the stimulation chamber, when compared to MPAvgat-vPAG axon terminal stimulation (n = 7; n = 4 males; n = 3 females); ∗p < 0.05. Error bars represent SD. [file Image_4.JPEG]
